# Inhibiting the inflammasome with MCC950 counteracts muscle pyroptosis and improves Duchenne muscular dystrophy

**DOI:** 10.3389/fimmu.2022.1049076

**Published:** 2022-12-07

**Authors:** Nicolas Dubuisson, María A. Davis-López de Carrizosa, Romain Versele, Camille M. Selvais, Laurence Noel, P. Y. D. Van den Bergh, Sonia M. Brichard, Michel Abou-Samra

**Affiliations:** ^1^ Endocrinology, Diabetes and Nutrition Unit, Institute of Experimental and Clinical Research (IREC), Medical Sector, Université Catholique de Louvain (UCLouvain), Brussels, Belgium; ^2^ Neuromuscular Reference Center, Department of Neurology, Cliniques Universitaires Saint-Luc, Brussels, Belgium; ^3^ Departamento de Fisiología, Facultad de Biología, Universidad de Sevilla, Sevilla, Spain

**Keywords:** Duchenne muscular dystrophy (DMD), MCC950, NLRP3 inflammasome, pyroptosis, muscle inflammation, gasdermin, N-GSDMD

## Abstract

**Background:**

Duchenne muscular dystrophy (DMD) is the most common inherited human myopathy. Typically, the secondary process involving severe inflammation and necrosis exacerbate disease progression. Previously, we reported that the NLRP3 inflammasome complex plays a crucial role in this disorder. Moreover, pyroptosis, a form of programmed necrotic cell death, is triggered by NLRP3 *via* gasdermin D (GSDMD). So far, pyroptosis has never been described either in healthy muscle or in dystrophic muscle. The aim of this study was to unravel the role of NLRP3 inflammasome in DMD and explore a potentially promising treatment with MCC950 that selectively inhibits NLRP3.

**Methods:**

Four‐week‐old mdx mice (*n*=6 per group) were orally treated for 2 months with MCC950 (mdx‐T), a highly potent, specific, small-molecule inhibitor of NLRP3, and compared with untreated (mdx) and wild-type (WT) mice. *In vivo* functional tests were carried out to measure the global force and endurance of mice. *Ex vivo* biochemical and molecular analyses were performed to evaluate the pathophysiology of the skeletal muscle. Finally, *in vitro* tests were conducted on primary cultures of DMD human myotubes.

**Results:**

After MCC950 treatment, mdx mice exhibited a significant reduction of inflammation, macrophage infiltration and oxidative stress (-20 to -65%, *P*<0.05 *vs* untreated mdx). Mdx‐T mice displayed considerably less myonecrosis (-54%, *P*<0.05 *vs* mdx) and fibrosis (-75%, *P*<0.01 *vs* mdx). Moreover, a more mature myofibre phenotype, characterized by larger-sized fibres and higher expression of mature myosin heavy chains 1 and 7 was observed. Mdx-T also exhibited enhanced force and resistance to fatigue (+20 to 60%, *P*<0.05 or less). These beneficial effects resulted from MCC950 inhibition of both active caspase-1 (-46%, *P=*0.075) and cleaved gasdermin D (N-GSDMD) (-42% in medium-sized-fibres, *P*<0.001). Finally, the anti-inflammatory action and the anti-pyroptotic effect of MCC950 were also recapitulated in DMD human myotubes.

**Conclusion:**

Specific inhibition of the NLRP3 inflammasome can significantly attenuate the dystrophic phenotype. A novel finding of this study is the overactivation of GSDMD, which is hampered by MCC950. This ultimately leads to less inflammation and pyroptosis and to a better muscle maturation and function. Targeting NLRP3 might lead to an effective therapeutic approach for a better management of DMD.

## Introduction

Duchenne muscular dystrophy (DMD) is the most common inherited myopathy and one of the most severe muscle-wasting diseases eventually leading to wheel chair, assisted ventilation and premature death (1). DMD is caused by mutations in the gene encoding dystrophin, a key scaffolding protein that provides structural stability and integrity to muscle fibre membrane. Dystrophin-deficient fibres are highly susceptible to injury, resulting in endless cycles of muscle necrosis and repair that lead to fibrosis and weakness (2). Although dystrophin mutations represent the primary cause of DMD, it is the secondary processes involving persistent inflammation and necrosis that likely exacerbate disease progression (3, 4).

The NLRP3 inflammasome plays a pivotal role in diseases resulting from sterile and excessive inflammatory responses (5, 6). Upon activation, NLRP3, a sensor of the innate immune system that belongs to the NOD-Like receptor family (NLR), oligomerizes with an adaptor (ASC) forming intracellular signalling hubs that activate caspase-1. Caspase-1 then cleaves two pro-inflammatory cytokines into their mature forms (IL-1β and IL-18) and gasdermin D (GSDMD) into its active effector. Next, released GSDMD generates pores in the plasma membrane, thereby leading to pyroptosis, a necrotic cell death type (5, 7) and ultimately facilitating the release of mature inflammatory cytokines. We have shown that the inflammasome is present within myofibres and is overactivated in dystrophic mouse muscle as well as in myotubes from DMD patients. Moreover, genetic inactivation of Nlrp3 markedly attenuates the dystrophic phenotype in mdx mice, a murine model of DMD (3). Targeting the inflammasome through highly potent, selective, small molecule inhibitors could thus be a promising approach for such a devastating disease.

The recently discovered molecule MCC950, is an extremely potent and selective inhibitor of NLRP3 (8–10). It proved to be successful in animal models characterised by some degree of sterile inflammation like atherosclerosis, colitis, cerebral ischemia or cholestatic liver injury (11–14). It also attenuated some biological and clinical features in a mouse model exhibiting a very rare syndrome due to an aberrant host Valosin-Containing Protein (VCP) and combining inclusion body myopathy with bone and cerebral lesions (15). However, in this model, the improvement in some organs may have indirect repercussions on the others. As yet, pharmacological blockade of the inflammasome has not been investigated in DMD, although NLRP3 plays a key pathogenic role in this muscular disease.

The aim of this work was to explore whether inflammasome blockade by MCC950 may play a beneficial role in DMD. To this end, MCC950 was orally administered for a period of 2 months, very early in mdx mice because muscle degenerative-regenerative cycles begin as soon as 3–4 weeks of age (16). We first examined whether treated mdx mice exhibited improved muscular function together with lower degree of skeletal muscle inflammation, oxidative stress, necrosis and fibrosis. We then unraveled the mechanisms underlying the potential beneficial effects brought about by MCC950. Finally, we examined whether some of these effects may be recapitulated in human by using primary cultures of DMD myotubes.

## Materials and methods

### Animals

C57BL/10ScSn-*Dmd^mdx^* J mdx mice (murine model of DMD) and C57BL/10ScSnJ mice [used as wild-type (WT) controls] were purchased from Jackson Laboratory (Maine, USA). Two small cohorts of mice were used, each cohort was divided into three groups of male mice. Once both cohorts were pooled, each group contained 6 mice. At 4 weeks of age, mice across all groups received solid drinks, replacing the water bottles. The first group was WT mice, the second group consisted of untreated mdx mice (mdx), while the third group was composed by treated mdx mice (mdx-T). This last group received MCC950 (40 mg/kg/day for 4 weeks, then 80 mg/kg/day for another 4-week period) (Avistron chemistry, Cornwall, UK) added to a solid drink, which was replaced every day. The choice of MCC950 dosage and duration was based on current literature and our preliminary experiments (15, 17). Animals were maintained under a standard laboratory chow and housed at a constant temperature (22°C) with a fixed 12 h light to 12 h dark cycle (lights on from 7 a.m. to 7 p.m.). 12-week-old mice were sacrificed between 09.00 and 11.00 h. Pairs of *tibialis anterior* (TA) and *quadriceps* (Q) muscles were weighed, frozen in liquid nitrogen, and stored at −80°C for subsequent analyses.

### *In vivo* studies of global force and resistance

At week 11 (i.e., 1 week prior to sacrifice), mice were submitted to two widely used and reliable functional tests (2, 3, 18).

*Wire test.* Mice were suspended by their limbs from a wire and the time until they completely released their grasp and fell was recorded. Mice that reached a set limit of 600 seconds were allowed to stop the experiment, while others were directly retested, up to three times, and their maximum hanging time was recorded. The holding impulse (body mass x hang time), used to oppose the gravitational force, was then calculated (18).

*Grip test.* Limb strength was recorded using a grid connected to a sensor (Panlab-Bioseb, Vitrolles, France). Mice were gently laid on the top of a grid so that their front paws could grip the grid. Mice were then pulled back steadily until the grip was released down the complete length of the grid. Each test was repeated three times at an interval of 15 min. Results are presented as the mean of the three values of force recorded, related to body weight (2).

### Bright-field histochemistry

*Tibialis anterior* muscles (TA) were fixed in 10% formalin for 24 h and embedded in paraffin. Immunohistochemistry was carried out as previously described (2) using antibodies directed against cluster of differentiation 68 (CD68), 4-hydroxy-2-nonenal (HNE), interleukin 1-beta detecting mature and precursor form (IL-1β), interleukin 18 detecting only mature form (IL-18), peroxiredoxin 3 (PRDX3), and tumor necrosis factor alpha (TNFα) (**Supplementary Information, Table S1**). Whole muscle sections were scanned, and then the optical density of diaminobenzidine (DAB, ThermoFisher-Scientific, Waltham, MA, USA) deposits (IL‐1β, TNFα, HNE and PRDX3) or the percentage of stained areas (IL-18, CD68) was quantified using Fiji (NIH, Maryland, USA). TA sections were also stained with haematoxylin and eosin. In addition, *Quadriceps* (Q) sections (described below) were stained with Picro-Sirius red (Abcam, Cambridge, UK) to evaluate muscle fibrosis. Fibrotic tissue was scored setting a color balance threshold and data obtained were expressed as the percentage of total section area.

### Immunofluorescence and morphometry

*Quadriceps* (Q) muscles were embedded in optimum cutting temperature medium (OCT; VWR International, Dublin, Ireland) and frozen in liquid nitrogen chilled isopentane (Sigma-Aldrich, St. Louis, MO, USA). 10 μm transversal cryosections were fixed with 4% paraformaldehyde and blocked with 10% goat serum. Antibodies directed against CD68, CD206, IL-1β, laminin, embryonic myosin heavy chain 3 (Myh3), and the cleaved form of gasdermin (N-GSDMD) (19), were used. Mouse IgG-AF488 was used for the detection of necrotic myofibres (**Table S1**). Secondary antibodies were AF488 and AF647-conjugated goat anti-rabbit or anti-rat (Sigma-Aldrich), respectively. Finally, nuclei were stained with DAPI (ThermoFisher-Scientific). Negative controls were performed by omission of the primary antibody (see **Supplementary Figure S1** as an example). Images were acquired with an epifluorescence (AXIO-observerZ1, Zeiss, Germany) or a confocal microscope (LSM800, Zeiss) for N-GSDMD, and staining was quantified with Fiji.

Total number of macrophages was quantified as the number of CD68 stained particles per field. Briefly, a threshold (1500, 65535) was applied for the CD68 channel on the 16-bit images with FIJI. Then, using the FIJI “Analyze Particles” tool, the number of particles/field above that threshold was counted. The proportion of M2 over the total number of macrophages was assessed calculating the ratio of CD68-CD206 double positive cells over CD68-only positive cells as described (20). IL-1β was quantified as the mean grey value of muscle section after background subtraction, and IgG uptake as the percentage of stained area per field after applying a fixed detection threshold. Myh3^+^ fibres were counted and expressed as percentage of the total number of fibres per field. N-GSDMD density (number of N-Gasdermin^+^ particles per 100 μm^2^) was quantified for each fibre and evaluated according to fibre size (see below). Totally 396, 1337 and 1378 fibres were analysed for WT, mdx and mdx-T groups respectively. Difference in fibres analysed between WT and the other two groups was explained by the higher number of small-sized fibres in dystrophic muscles (see later).

Fibre size assessed by cross-sectional area (CSA) and minimum Feret’s diameter (MFD), and centrally nucleated fibres (CNF) were calculated using a personalized version of MuscleJ (21) based on the automatic detection of laminin and DAPI immunofluorescence. Approximately 6500 fibres per group were quantified. Fibres were then assigned to one of three categories based on their size [small (S), medium (M) or large (L)]. Middle-sized fibres were defined as ranging from the 10^th^ to the 90^th^ percentile (P) of WT fibre size distribution, whereas other fibres were categorized as small (below P10; <33µm) and large (above P90; >74µm), respectively.

### Culture of human myotubes

Primary cultures of human skeletal muscle cells were initiated from myoblasts of DMD patients (n = 4; age range: 12–15 years) and healthy subjects (n = 3; age range: 15–17 years), which were provided by the French Telethon Myobank-AFM. Myoblasts were grown in DMEM/F-12 supplemented with 20% fetal bovine serum (FBS), 1% L-Glutamine, 1% non-essential amino acids and 1% penicillin-streptomycin (all from ThermoFisher-Scientific) at 37°C in 5% CO2. After reaching a density of 80-90%, the growth medium was replaced with a fusion medium, where 20% FBS was replaced by 2% horse serum, and differentiation was allowed to continue for 14 days (time required to obtain mature myotubes with characteristic elongated and multinucleated morphology). Medium was changed every other day. Cells were always used at passages between 4 and 10. At day 14, myotubes were pre-treated or not with MCC950 (10 μM) for 24 h, while being challenged or not with human recombinant TNFα (15 ng/mL) + human interferon gamma (IFNγ) (15 ng/mL) (both from PeproTech, New Jersey, USA) for the last 22 h and then stimulated with ATP (5mM) (Roche, Basel, Switzerland) for the last 2 h.

### RNA extraction and real-time quantitative PCR

RNA was isolated from muscle tissues or from cultured cells with TriPure reagent (Sigma-Aldrich). RT-qPCR primers for mouse cyclophilin, NLRP3 and ASC were used as previously reported (3). New mouse primer sequences were transforming growth factor beta (TGFβ) (**Table S2**). RT-qPCR primers for human TATA box-binding protein (TBP), TNFα, and IL-1β, were also similar to those previously reported (22). New human primer sequences were IL-18 (**Table S2**). Threshold cycles (Ct) were measured in duplicate.

### Protein extraction

Muscle samples and cultured cells were homogenized in a lysis buffer supplemented with 1% protease/phosphatase inhibitor cocktail (both from Cell Signaling Technology, Leiden, The Netherlands) and 10 mM NaF (Sigma-Aldrich). Protein levels were quantified using the Bradford method and 10–150 μg of total protein extracts were used for each analysis.

### ELISAs

ELISA assays were used to specifically detect and quantify Utrophin A (UTRN) (Antibodies Online, Atlanta, USA), the active forms (phosphorylated form) of Smad2, of p65 subunit of nuclear factor-kappa B (NF-κB) (both from Cell Signaling Technology) and of N-GSDMD (MyBiosource, San Diego, USA). Kits were based on colorimetric methods and were carried out following manufacturer’s instructions.

### Western blots

Immunoblotting was performed, as reported (3), by using a rabbit polyclonal antibody directed against full-length caspase-1 (46 kDa) and cleaved caspase-1 (20 kDa) (Bio-connect, Huissen, The Nederlands). Signals were revealed by enhanced chemiluminescence, then quantified and normalized to total proteins of each corresponding lane detected with Ponceau staining (**Supplementary Figure S2**) (23), using Fiji.

### Statistical analysis

Results are means ± standard error of mean (SEM) for the indicated number of mice or human subjects. When the three groups of mice (WT, mdx, and mdx-T) were compared, the effects of MCC950 were assessed by one-way analysis of variance followed by Tukey’s test. When four cell cultures conditions were compared, the effects of MCC950 and inflammation were assessed by two-way analysis of variance (raw paired data for each subject) followed by *post hoc* Sidak’s multiple comparisons. All statistical analyses were performed with Prism 9 (GraphPad Software, Inc., San Diego, CA). Differences were considered statistically significant at *P* < 0.05.

## Results

### MCC950 does not affect muscle mass while it improves muscle function

MCC950 was administered orally to mdx mice for 2 months, starting at 4 weeks of age. Mdx littermates were thus separated into two groups: those who received a daily dose of MCC950 (mdx-T) and those who were left untreated (mdx). Both groups were also compared with untreated wild-type (WT) control mice. MCC950 treatment did affect neither the total body weight of dystrophic mice, nor the weight of removed muscles (*Tibialis anterior* (TA) and *Quadriceps* (Q)) (**Supplementary Figure S3**). Macroscopic evaluation of liver and kidney after treatment was also unremarkable.

**Figure 1:**
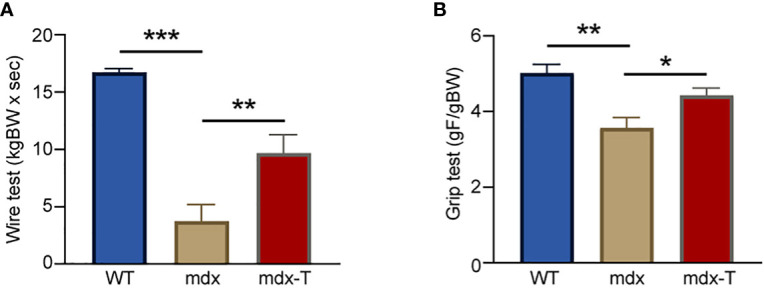
MCC950 treatment improves global force and resistance of mdx mice. Three groups of mice were compared at the age of 11 weeks: WT, mdx (untreated) and mdx-T (mdx treated with MCC950) mice. Functional tests were carried out *in vivo*. **(A)** Mice were subjected to a wire test where they were suspended by their limbs and the time until they completely released the wire and fell was registered (seconds). This time was then normalized to body weight (kgBW x sec). **(B)** Mice were lowered on a grid connected to a sensor to measure the muscle force of their forelimbs; data were then expressed in gram-force relative to gram-body weight (gF/gBW). Data are means ± SEM; *n* = 6 mice per group for all experiments. Statistical analysis was performed using one-way ANOVA followed by Tukey’s test. ^*^ *P* < 0.05, ^**^ *P* < 0.01, ^***^ *P* < 0.001.

In order to evaluate the effects of MCC950 on muscle function, mice were subjected to two functional tests *in vivo*: the wire test and the grip test. The wire test gives an indication on muscle force and resistance to fatigue. In this test, the time during which the mouse is suspended on a horizontal wire is measured. Mdx mice fell down faster than WT mice, while mdx-T ones showed intermediate resistance (+60% *vs* mdx) (**Figure 1A**). The grip test measures strength of limb muscles. The force developed by forelimbs was lower in mdx mice than in WT ones, while it was partially rescued by MCC950 treatment (+20% *vs* mdx) (**Figure 1B**). These data indicate enhanced force and resistance to fatigue in dystrophic muscle under MCC950 treatment.

### MCC950 effectively reduces muscle inflammation and oxidative stress

**Figure 2:**
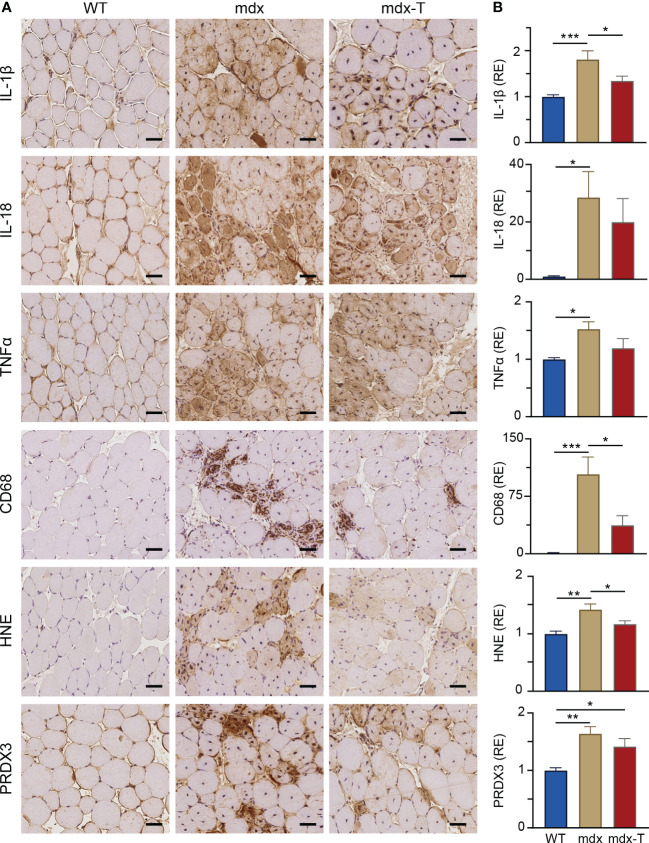
MCC950 treatment reduces muscle inflammation and oxidative stress in mdx mice. Immunohistochemistry was performed on *Tibialis anterior* muscle (TA) of the 3 groups of mice. **(A)** Sections were stained with specific antibodies directed against three pro‐inflammatory cytokines (IL-1β, IL-18 and TNFα), one pan-macrophage marker (CD68) and two oxidative stress markers (HNE and PRDX3). Representative sections for 6 mice per group are shown. Scale bars = 50 μm. **(B)** Quantification of inflammation and oxidative stress markers. Data are presented as relative expression (RE) compared to WT values and are means ± SEM; *n* = 6 mice per group for all experiments. Statistical analysis was performed using a one‐way ANOVA followed by Tukey’s test. ^*^ *P* < 0.05, ^**^ *P* < 0.01, ^***^ *P* < 0.001.

We tested the hypothesis that oral administration of MCC950 could slow down the progression of the dystrophic pathology by counteracting excessive inflammatory and oxidative reactions. When compared to WT mice, myofibres from TA muscles of mdx mice displayed a strong immunolabeling for the inflammatory cytokines, IL-1β and IL-18 (**Figure 2A**). Quantification of DAB staining confirmed that IL-1β and IL-18 immunolabeling was much higher in mdx than in WT mice (**Figure 2B**). Qualitatively, similar results were observed for TNFα, another main inflammatory cytokine and for CD68, a pan-macrophage marker. Accordingly, massive inflammatory infiltrates were observed within dystrophic muscle after haematoxylin and eosin staining (**Supplementary Figure S4**). Like pro-inflammatory markers, two oxidative stress markers (HNE, a lipid peroxidation product and PRDX3, an antioxidant enzyme), were increased in mdx mice.

In mdx-T mice, all these markers of inflammation or oxidative stress were either significantly decreased compared to regular mdx (IL-1β: -25%; CD68: -64%; HNE: -18%) or were not (or less) significantly different from those of WT ones (**Figure 2A, B**).

### MCC950 slows down the necrosis-regeneration turnover in dystrophic muscle

**Figure 3:**
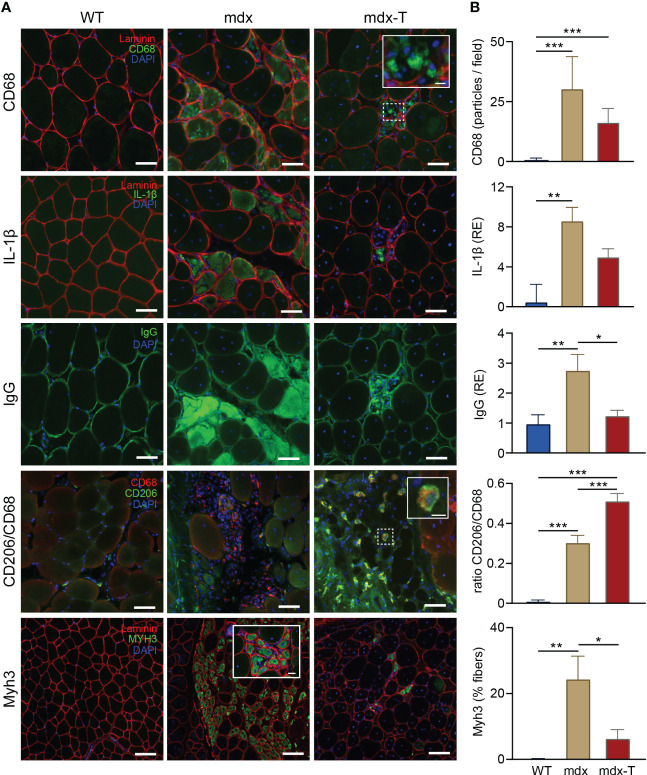
MCC950 treatment curbs the accelerated necrosis-regeneration turn-over of mdx mice. Immunofluorescence staining was performed in *Quadriceps* muscle (Q) from the 3 groups of mice. **(A)** Sections were stained with specific antibodies directed against CD68, IL‐1β, mouse IgG, CD206 and MyH3 (all in green). Laminin antibody was used to delineate basal membrane (red). Nuclei were counterstained with DAPI (blue). The first three immunolabellings (CD68, IL1-β and IgG) were performed on serial cross sections. Scale bars = 50 μm (CD68, IL-1β, IgG and CD206) and 100 µm (Myh3). Insets: higher magnification of CD68^+^ and CD206^+^ macrophages (scale bars = 10µm) and Myh3^+^ fibres (scale bars = 20 µm). **(B)** Quantification of CD68, IL‐1β, mouse IgG, CD206 and MyH3. Results for CD68 and Myh3 are shown as number of positive particles and % of positive fibres, respectively. IL1-β and IgG uptake are presented as relative expression (RE) compared to WT values. CD206 is represented as the ratio of CD206+/CD68+. Data are means ± SEM; *n* = 6 mice per group for all experiments. Statistical analysis was performed using one‐way ANOVA followed by Tukey’s test. ^*^ *P* < 0.05, ^**^ *P* < 0.01, ^***^ *P* < 0.001.

We next tested the idea that MCC950 could curb the necrosis-regeneration turnover, which is otherwise accelerated in the dystrophic muscle. For these experiments, we performed immunofluorescence on serial Q sections for the three first labellings (**Figure 3A** and **Supplementary Figure S1**). As in TA (see **Figure 2**), mdx mice showed a strong immunostaining for CD68 (detected as stained particles) and for IL-1β, both of which tended to decrease in mdx-T animals (**Figure 3B**). Likewise, the percentage of necrotic areas assessed by the uptake of mouse IgG, a well-known marker of membrane leakage (24), was markedly increased in mdx mice but was reduced to WT values in mdx-T ones (**Figure 3B**). Moreover, areas that were positive for CD68 and IL-1β were also positive for IgG, indicating an interplay between macrophage infiltration, IL-1β production and necrosis that clusters in the same muscle subareas.

In other Q sections, we also showed that among CD68 positive macrophages, the portion of CD206 positive cells, a specific marker of M2 macrophages (25), was significantly increased in mdx-T mice in comparison with untreated mice. This indicates a potential switch in macrophage polarisation towards an anti-inflammatory phenotype (**Figure 3A**).

We next quantified the expression of embryonic myosin heavy chain 3 (Myh3), a marker of early muscle regeneration (26). This myosin type is usually expressed during development but can be transiently re-expressed in adult muscle upon injury (26). As expected, there was almost no embryonic positive myofibres in WT mice, while mdx mice displayed approximately 25% of immuno-positive fibres, a percentage that decreased to <10% in treated animals (**Figure 3A, B**).

Taken together, these data indicate that mdx mice displayed large inflammatory and necrotic areas combined with early embryonic regenerating myofibres. All of which were markedly attenuated under treatment.

### MCC950 treatment enhances muscle fibre size and maturation in mdx mice

**Figure 4:**
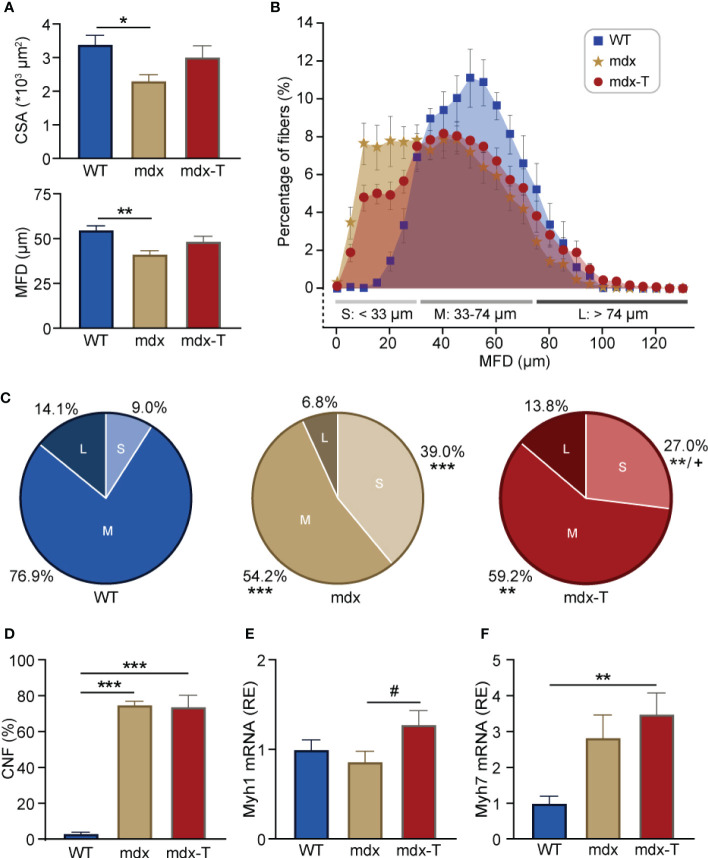
MCC950 treatment enhances muscle fibre size and maturation in mdx mice. **(A)** Quantification of global fibre size based on cross-sectional area (CSA) or minimum Feret’s diameter (MFD) in *Q* sections from the 3 groups of mice. **(B)** Overall distribution of fibre MFD. Data are expressed as percentage of myofibres in bins of 5 µm each. Fibres were classified into three categories based on their size [small (S), medium (M) or large (L)]. **(C)** Pie chart representing the percentage of small (pale), middle-sized (regular) and large myofibres (dark) for each group. **(D)** Quantification of the percentage of central nucleated fibres (CNF) per field. **(E, F)** mRNA levels of Myh1 and Myh7, markers of glycolytic and oxidative muscle fibres, respectively. These levels were normalized to cyclophilin A, and subsequent ratios were presented as relative expression compared to WT values. Data are means ± SEM; *n* = 6 mice per group for all experiments. Statistical analysis was performed using one-way ANOVA followed by Tukey’s test **(A- F)** ^*^ *P* < 0.05, ^**^ *P* < 0.01, ^***^ *P* < 0.001, ^+^ *P <*0.05 (comparison between small fibres from mdx and mdx-t mice) **(C)**, ^#^ *P* = 0.08 **(E)**.

In line with the observation that newly regenerating fibres are small in size (27), average myofibre size measured either CSA or MFD was decreased in mdx mice but was not different in mdx-T animals compared to WT ones (**Figure 4A**). Moreover, fibre distribution based on MFD indicates that the percentage of small (S) myofibres was higher in mdx than in WT mice (**Figure 4B**), with a significant reduction in mdx treated mice compared to regular mdx (**Figure 4C**, Pale red *vs* pale beige pie charts).

However, the proportion of fibres with central nuclei (CNF), a hallmark of fibres that have undergone regeneration, which was almost undetectable in WT mice, remained similarly elevated in mdx mice whether treated or not (**Figure 4D**). Yet, this marker does not discriminate between fibres at the early or late stage of regeneration (28). Because mdx-T mice have globally larger fibres than regular mdx (**Figure 4A**), with less staining for Myh3, we speculated that the regeneration process was more complete under MCC950. We thus measured the expression of mature forms of myosin heavy chain such as Myh1 and Myh7. Accordingly, mRNA abundance of Myh1, a marker of fast-twitch glycolytic type II fibres, tended to be increased in mdx-T mice compared to untreated ones (+32%; **Figure 4E**). Likewise, Myh7, a marker of slow-twitch oxidative type I fibres, was augmented in mdx-T mice compared to control WT (3.5-fold), while there was no significant difference between regular mdx and WT mice (**Figure 4F**).

Taken together, these results indicate that mdx-T mice exhibited a significant reduction of necrotic areas requiring fewer regenerating fibres, which develop a more mature phenotype.

### MCC950 attenuates fibrosis in mdx mice

**Figure 5:**
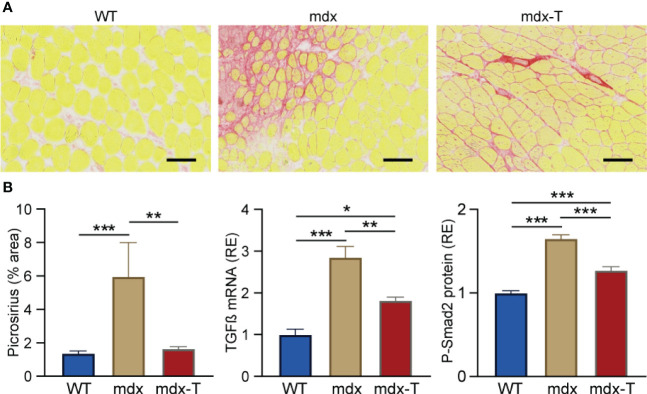
MCC950 treatment markedly reduces muscle fibrosis in mdx mice. Experiments were performed on *Q* from the three groups of mice. **(A)** Picro-Sirius red staining. **(B)** Quantification of picro-Sirius red staining (expressed as % area). Scale bars = 200 μm. mRNA levels of TGFβ, a marker of extracellular matrix production. These levels were normalized to cyclophilin **(A)** ELISA assay was used to quantify phosphorylated-Smad2 (P-Smad2). Results for TGFβ and P-Smad2 were then presented as relative expression compared to WT values. Data are means ± SEM; *n* = 6 mice per group for all experiments. Statistical analysis was performed using one-way ANOVA followed by Tukey’s test. ^*^ *P* < 0.05, ^**^ *P* < 0.01, ^***^ *P* < 0.001.

Because inflammation and fibrosis can be viewed as a continuum of events within the framework of tissue defence, repair and regeneration, we next tested whether oral administration of MCC950 could decrease fibrosis in dystrophic muscle. Mdx muscle displayed a strong staining for Picro-Sirius red, a marker of fibrosis labelling collagen I and III. Yet, the percentage of fibrotic areas was reduced by ~75% in mdx-T mice (**Figure 5A, B**).

In addition, mRNA levels of TGFβ, a highly pleiotropic cytokine playing an important role in wound healing and inducing the production of extracellular matrix, were increased in mdx mice, while these levels were decreased by ~35% in mdx-T ones (**Figure 5B**).

We then measured Phosphorylated-Smad2 (P-Smad2), an effector protein being part of the TGFβ pathway, which is also a marker of muscle fibrosis. Likewise, mdx mice showed higher P-Smad2 protein levels in comparison with WT mice. Conversely, these levels were significantly diminished in mdx-T mice (~25%) (**Figure 5B**).

Taken together, these results indicate that mdx-T mice displayed a significant reduction of factors responsible for the fibrotic transition of myofibres, suggesting an anti-fibrotic potential for MCC950.

### MCC950 exerts its effect by acting on key effectors of the inflammasome complex and on utrophin A, in mdx mice

On activation, NLRP3 oligomerizes with ASC, forming intracellular signalling hubs that activate caspase-1. Caspase-1 then generates the mature forms of pro-inflammatory cytokines (IL-1β and IL-18) and cleaves GSDMD, triggering pyroptosis. As MCC950 directly inactivates NLRP3 (9, 10), we explored its action on the two main effectors of the inflammasome complex: active Caspase-1 and N-GSDMD.

**Figure 6:**
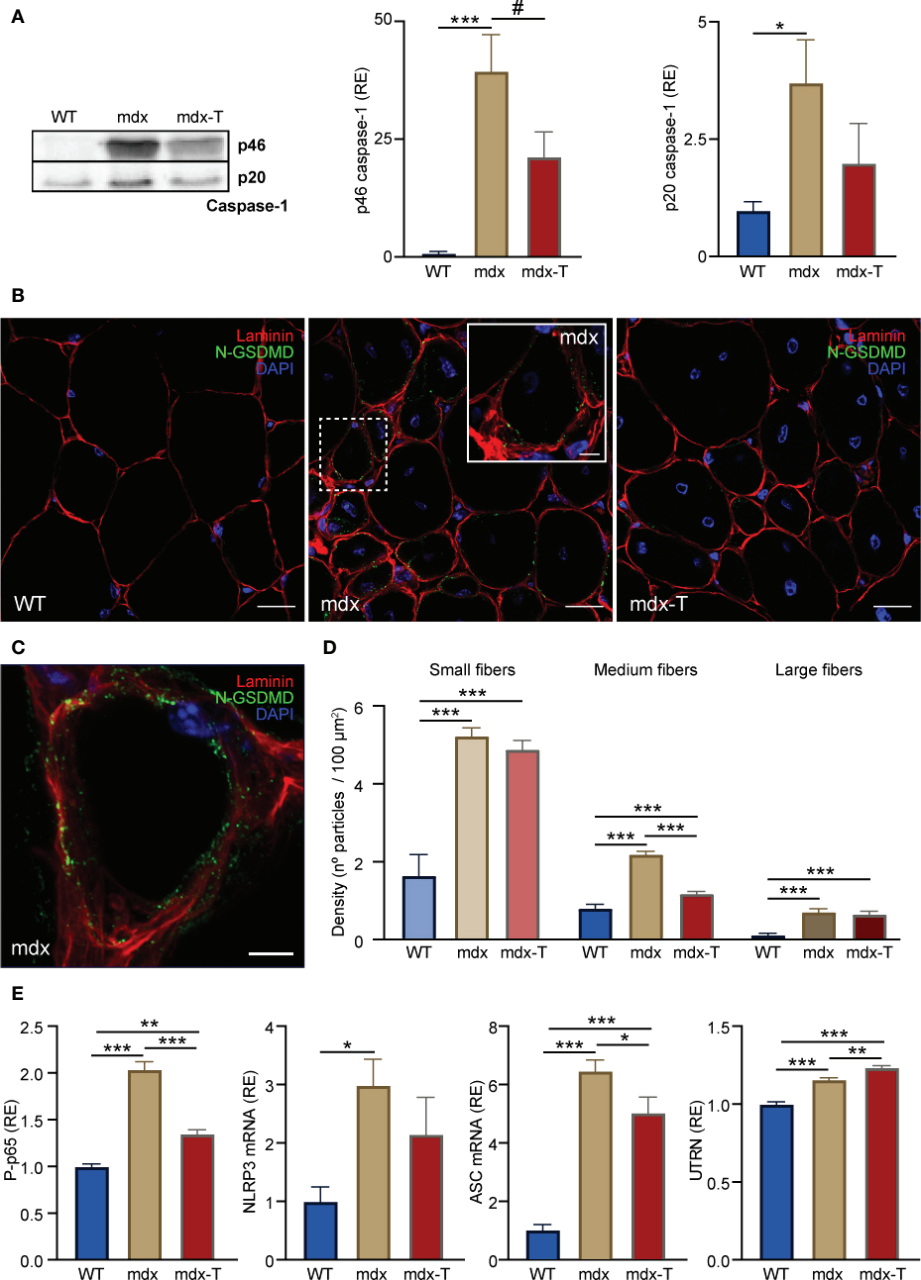
MCC950 treatment disrupts pyroptosis and increases the levels of a dystrophin analogue in mdx mice. Experiments were performed on *Q* from the three groups of mice. **(A)** Caspase-1 analysis by Western blotting. Levels of each marker (full-length p46 and cleaved p20 subunit) were normalized to Ponceau (shown in Figure S1) and expressed relative to WT values. **(B)** Distribution of N-GSDMD density by immunofluorescence labelling. Sections were stained with specific antibodies directed against N-GSDMD (green) and laminin (red) and co-stained with DAPI (blue). The inset (dashed white square) shows the labelling of N-GSDMD in a small-sized fibre of a mdx untreated animal. **(C)** 3D reconstruction of a small mdx fibre showing the distribution of N-GSDMD (green) close to the sarcolemma (laminin, red). Scale bars: B = 20 μm, inset on B = 5 μm, C = 5 μm. **(D)** N-GSDMD density (number of stained particles per 100 µm²) was quantified for each fibre and presented according to fibre size. **(E)** Levels of NF-κB activity, NLRP3 and ASC mRNAs, and UTRN protein. ELISAs were used to quantify the active phosphorylated form of the p65 subunit of NF-κB (P-p65) and UTRN. Absorbance data are presented as relative expression (RE) compared with WT values. mRNA levels were normalized to cyclophilin A, and the subsequent ratios presented as relative expression compared with WT. Data are expressed as means ± SEM; *n* = 6 mice per group for all experiments. Statistical analysis was performed using one‐way ANOVA followed by Tukey’s test. ^*^ *P* < 0.05, ^**^ *P* < 0.01, ^***^ *P* < 0.001 ^#^ *P* = 0.081 **(A)**.

Firstly, we measured active Caspase-1 by Western blotting. The dominant subunit of active caspase-1 has recently been shown to be full-length p46 (29). Our results confirm that p46 was strikingly elevated in mdx mice while it was reduced by ~45% in treated animals. Its cleaved product p20 follows a similar pattern with a rise in mdx mice and a ~45% decrease (though not significant) under MCC950 (**Figure 6A** and **Supplementary Figure S2**).

Secondly, we studied the active form of GSDMD (i.e. its cleaved N-GSDMD fragment), a gold standard marker to detect pyroptosis (19). N-GSDMD was detected by immunofluorescence as stained green particles mainly located close to the sarcolemma. These particles were more abundant in mdx-mice (whether treated or not) than in WT ones (**Figure 6B, C**). N-GSDMD density was also inversely related to fibre size, being predominant in small and medium dystrophic myofibres while being almost absent in larger ones. Mdx-T mice exhibited a significant reduction of N-GSDMD density in medium sized-fibres compared to regular mdx, thereby suggesting a reduction of pyroptosis (**Figure 6D**).

As reduced action of caspase-1 and N-GSDMD should in turn reduce the activity of the transcription factor NF-κB, a master regulator of inflammation, we measured this parameter under MCC950 treatment. As expected, the active phosphorylated form of the p65 subunit of NF-κB was increased about two-fold in mdx mice, while it was reduced by 34% in mdx-T ones (**Figure 6E**). NF-κB serves as a priming signal that upregulates several inflammasome components. Accordingly, mRNA levels of NLRP3 were drastically increased in mdx mice and showed a tendency towards reduction in treated animals (**Figure 6E**). Likewise, mRNA levels of ASC were decreased after treatment (**Figure 6E**). Eventually, we measured Utrophin A (UTRN), an analogue of dystrophin that can help rescue the dystrophic phenotype (2, 30). Accordingly, UTRN protein levels were slightly augmented in mdx mice, likely to compensate for the lack of dystrophin. These levels were further increased in mdx-T mice, possibly as a result of lower inflammation (31) (**Figure 6E**).

### MCC950 recapitulates its beneficial effect on human myotubes challenged by pro-inflammatory cytokines

Due to its convenience and cost-effectiveness, the mdx mouse remains the most widely used model for studying DMD (32). However, since this model exhibits a milder phenotype than patients, it was important to validate our data in human myotubes. We thus examined the direct effects of MCC950 in primary cultures of DMD human myotubes. In order to mimic the inflammatory microenvironment of DMD, myotubes were challenged with an inflammatory stimulus (TNFα/IFNγ), along with ATP, a pyroptosis enhancer, while being pre-treated or not with MCC950.

**Figure 7:**
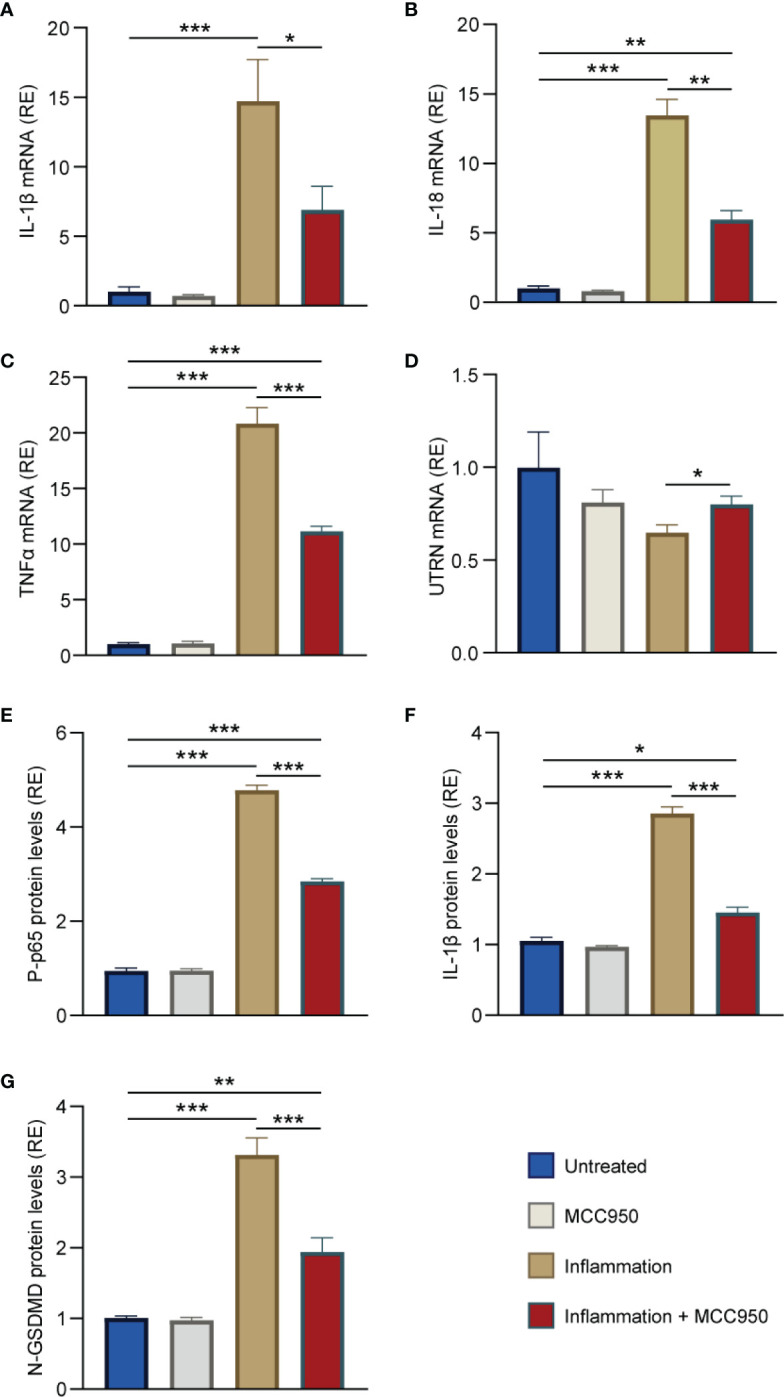
MCC950 treatment lessens inflammation and pyroptosis in challenged myotubes from DMD patients. **(A-D)** mRNA levels of IL-1β, IL-18, TNFα, and UTRN in primary cultures of myotubes obtained from DMD patients. mRNA levels were normalized to human TATA box-binding protein. **(E-G)** ELISAs were used to quantify the active phosphorylated form of the p65 subunit of NF-κB (P-p65), IL-1β and N-GSDMD. Absorbance data are presented as relative expression (RE) compared with WT values. Cells were pre-treated or not with MCC950 (10 μM) for 24 h, while being challenged or not with human recombinant TNFα (15 ng/mL) + human interferon gamma (IFNγ) (15 ng/mL) for the last 22 h and then stimulated with ATP (5mM) for the last 2 h. The subsequent ratios are presented as relative expression compared with basal conditions (i.e. no ATP, no inflammation and no MCC950, represented by blue columns). Data are means ± SEM for 4 cultures, each obtained from a different donor (i.e., 4 DMD subjects). Statistical analysis was performed on paired data using two-way analysis of variance followed by Sidak’s multiple comparison test. ^*^ *P* < 0.05, ^**^ *P* < 0.01, ^***^ *P* < 0.001.

We first tested the expression of the two pro-inflammatory cytokines, IL-1β and IL-18. mRNA levels for both cytokines were highly raised under inflammatory conditions, while MCC950 treatment reduced by ~50% their abundance (fourth *vs* third column) (**Figures 7A, B**). We also tested the expression of another inflammatory cytokine, TNFα, which is known to be significantly up-regulated in murine and human dystrophic muscles (2). As expected, the pattern of TNFα mRNA was very similar to that of IL-1β and IL-18 (**Figure 7C**). In addition, UTRN gene expression was decreased under inflammatory conditions, while MCC950 was capable of increasing its abundance by ~20% (fourth *vs* third column) (**Figure 7D**). Similar beneficial effects of MCC950 treatment were also observed on challenged human myotubes derived from healthy subjects (**Supplementary Figure S5**).

Moreover, the phosphorylated and active form of the p65 subunit of NF-κB (P-p65) was 5-fold higher in inflammatory conditions when compared to untreated ones (third *vs* first column), but was then down-regulated by ~40% under MCC950 treatment (fourth *vs* third column) (**Figure 7E**). Similarly, IL-1β protein levels were increased by 3-fold in inflammatory conditions while being halved after treatment (**Figure 7F**). Finally, DMD myotubes challenged with inflammation displayed high levels of activated N-GSDMD (>3-fold increase) (third *vs* first column). Again, treatment with MCC950 was able to halve those levels (**Figure 7G**).

Taken together, these results indicate a strong anti-inflammatory and anti-pyroptotic effects of MCC950 in human myotubes.

## Discussion

The inflammasome is involved in the initiation or progression of diseases with a high impact on public health, such as metabolic disorders and neurodegenerative diseases (3). It is also involved in some muscle diseases like dysferlin-deficiency (33) or aberrant host VCP (15). Over-expression and activation of the NLRP3 inflammasome also plays a crucial pathogenic role in DMD. Conversely, genetic disruption of Nlrp3 (3), but oddly not of ASC (34), alleviates the dystrophic phenotype of mdx mice. Herein, we found that therapeutic inhibition of the inflammasome exerts potent protective effects on the dystrophic skeletal muscle, thereby offering a new and exciting prospect for managing DMD.

**Figure 8:**
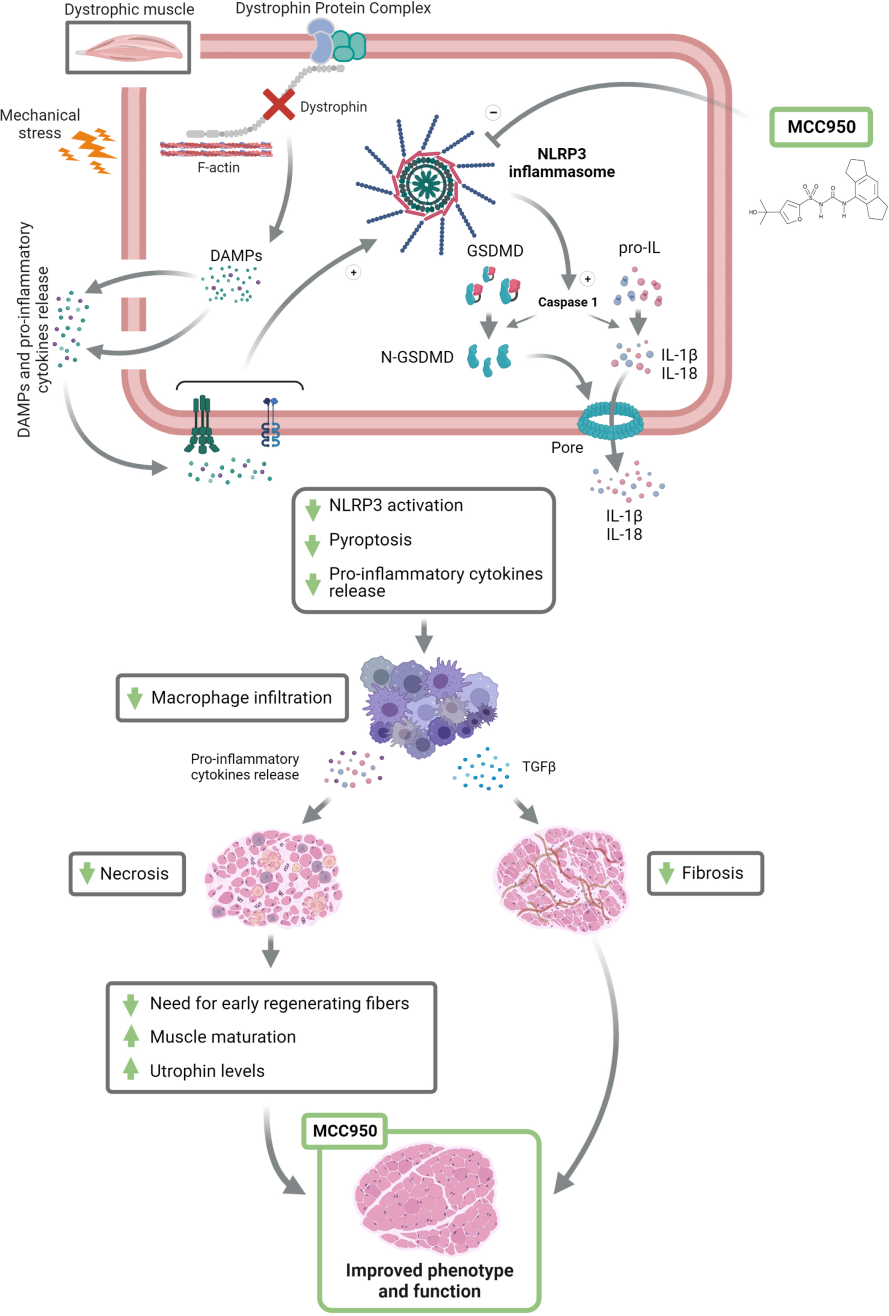
Inhibiting the inflammasome helps rescue the dystrophic phenotype*. DMD* gene mutations cause the absence of the dystrophin protein, which weakens the fibre membrane making it more sensitive to mechanical stress. This leads to microtears and the release of damage-associated molecular patterns (DAMPs) and pro-inflammatory cytokines. The latter will activate NLRP3 inflammasome and consequently caspase-1, which in turn cleaves and activates N-GSDMD, IL-1β and IL-18, leading to pyroptotic channel formation and the release of mature pro-inflammatory cytokines. By blocking NLRP3, MCC950 could markedly attenuate this process. This beneficial action will decrease macrophage (M1 and M2) infiltration and shift the macrophage polarization towards an anti-inflammatory phenotype (M2), leading to anti-necrotic and anti-fibrotic properties. Finally, MCC950 will allow for a better myofibre maturation and increase utrophin expression, therefore leading to an improved muscle function and phenotype. +, stimulation; -, inhibition; GSDMD, gasdermin D. This figure was created with Biorender.

Oral administration of MCC950 to mdx mice for 2 months decreased muscle inflammation as shown by the reduction of pro-inflammatory cytokines such as IL-1β and IL-18 (**Figure 8**). The total number of macrophages infiltrates (CD68+) was also reduced. Interestingly, the treatment seems to promote a shift towards a M2 oriented population attested by the increased CD206/CD68 ratio, which is relevant as M2 macrophages play a major role in anti-inflammatory responses (25). Accordingly, MCC950 treated animals also exhibited a reduction of necrosis and fibrosis (**Figure 8**) (24, 28). Less necrosis was then associated with less need for very early regenerating myofibres (26). Moreover, fibre maturation was more complete under MCC950, as attested by enhanced expression of mature forms of myosin heavy chain such as Myh1 and Myh7 and larger fibre size (**Figure 8**). In addition, MCC950 treatment potently attenuated muscle fibrosis, by reducing TGFβ levels and curbing its signaling pathway involving Smad2 protein (25). All these beneficial effects, together with a slight rise in utrophin may improve muscle function and attenuate the dystrophic phenotype.

We next explored the main mechanisms underlying the protective action brought about by MCC950 on dystrophic muscle. To this end, we investigated two key effectors of the inflammasome complex: Caspase-1 and GSDMD. Active Caspase-1 generates the mature forms of pro-inflammatory cytokines (IL-1β and IL-18) and cleaves GSDMD, triggering pyroptosis (**Figure 8**). Both full-length Caspase-1 (p46), which could be the dominant species of active caspase-1 (29) and its cleavage product (p20) were strongly increased in mdx mice but tended to be reduced under MCC950. The second effector, GSDMD is the executioner of pyroptosis *via* its cleaved N-terminal fragment (N-GSDMD). Pyroptosis is a form of programmed necrotic cell death caused by plasma membrane pore formation, resulting in cell swelling and eventually lysis. The pores can also serve as a gate for extracellular release of mature interleukins, resulting in an amplification of the inflammatory response (5, 7) (**Figure 8**). To the best of our knowledge, GSDMD has not yet been studied *in vivo* or *ex vivo* in skeletal muscle, and certainly not in dystrophic muscle. Herein, we show by immunofluorescence the location and density of N-GSDMD in dystrophic muscle and the effect exerted by MCC950. As expected, N-GSDMD was predominantly located in close contact to the fibre sarcolemma, where it forms the pyroptotic pores (5, 7, 19). Moreover, N-GSDMD particles were rarely detectable in healthy muscle, while being abundant in dystrophic one. These findings indicate that pyroptosis could be a novel programmed cell death type in DMD. Furthermore, density of the active form of GSDMD was inversely related to fibre size, suggesting that small and medium size myofibre are more prone to pyroptosis. This is in line with the recent hypothesis stating that branched dystrophic fibres, which are smaller in size, are very sensitive to rupture during muscle activity, leading to fibre death (35–37). Remarkably, mdx mice treated with MCC950 showed a significant reduction of N-GSDMD density in medium-sized myofibres compared to regular mdx. Although there were no significant differences in small-sized fibres between both groups, it should be stressed that the proportion of small fibres was lower in mdx-T than in regular mdx (27 *vs* 39%, **Figure 4C** pale red *vs* beige S marked pie charts). Taken together, these results indicate that the overall density of N-GSDMD is actually decreased in treated animals, thereby demonstrating an important effect of MCC950 on pyroptosis in the dystrophic muscle. Blockade of both main effectors of NLRP3 ultimately led to decreased activity of NF-κB, which in turn reduced the priming of NLRP3 (i.e., reduced transcription of several inflammasome components and lowered the expression of other pro-inflammatory actors like TNFα; see **Figures 2** and **7**) (38).

To summarise, by inhibiting the NLRP3 inflammasome, MCC950 strongly attenuates the activation of N-GSDMD, the release of pro-inflammatory cytokines, and pyroptosis in mdx mice. These beneficial effects reduce macrophage infiltration, thereby leading to less inflammation, necrosis, and fibrosis and consequently enhance muscle maturation, function, and phenotype (**Figure 8**).

We next transposed our mouse data in human, by using primary cultures of myotubes derived from either healthy or DMD subjects. As expected, we recapitulated the protective anti-inflammatory and anti-pyroptotic effects obtained in mice, strongly confirming the potential value of MCC950 in DMD patients.

DMD still awaits efficient gene therapy (39, 40). Meanwhile, glucocorticoids (GCs) remain the only option commonly used in this disease (1, 41). Their beneficial effects have mostly been ascribed to reduced inflammation (42). However, such a treatment is hampered by several adverse effects: weight gain, growth retardation, Cushingoid appearance as well as bone demineralization, glucose intolerance and hypertension. NLRP3 inhibitors could well be a strong alternative to GCs due to their powerful anti-inflammatory, anti-necrotic and anti-fibrotic action on the skeletal muscle. They also appear to be more selective than GCs, by only affecting the NLRP3 inflammasome pathway, thus implying less adverse events.

In addition, by targeting the NLRP3 inflammasome, MCC950 remains highly potent in comparison to specific inhibitors downstream of NLRP3 pathway. For instance, MCC950 was able to rescue neonatal lethality in a genetic mouse model of Cryopyrin-Associated Autoinflammatory Syndromes (CAPS) caused by NLRP3 activating mutation while targeted blockade of IL−1β alone was unable to do so (8, 43). This was ascribed to additional inhibition of IL-18 and pyroptosis afforded by MCC950. Moreover, specific targeting of NLRP3 (and not of the major anti-microbial inflammasomes NLRC4 and NLRP1) may explain why MCC950 is less immunosuppressive than biologic compounds (e.g., Canakinumab, which may clinically increase the risk of serious infections (8, 17).

Pharmaceutical companies are investing in specific inhibitors of NLRP3. Some of them are under investigation for inflammatory diseases such as Inzomelid for CAPS (phase I) (ClinicalTrials.gov Identifier: NCT04015076), OLT1177 for treatment of gout flares (phase II) (44), and ZYIL1 for viral inflammatory diseases characterised by cytokine IL-1β flare. (phase I) (ClinicalTrials.gov Identifier: NCT04972188). We feel that MCC950 and other specific inflammasome inhibitors that will turn out to be safe and successful in clinical trial, have a crucial role to play in managing DMD treatment.

## Conclusion

In summary, the innate immune system and the NLRP3 complex play a central role in the pathogenesis of DMD. We highlight GSDMD-induced pyroptosis as a novel type of programmed necrotic cell death in this disease. We undoubtedly demonstrate that MCC950, a small molecule inhibiting selectively NLRP3, can greatly attenuate the dystrophic phenotype in mdx mice, due to its anti-inflammatory, anti-pyroptotic and anti-fibrotic properties. Inflammasome inhibitors could open new therapeutic prospects for the management of DMD or other muscle and inflammatory disorders.

## Data availability statement

The raw data supporting the conclusions of this article will be made available by the authors, without undue reservation.

## Ethics statement

The studies involving human participants were reviewed and approved by Bioethics department of the Direction Générale de la Recherche et de l’Innovation, France (DGRI no. AC-2013-1868). Written informed consent to participate in this study was provided by the participants’ legal guardian/next of kin.The animal study was reviewed and approved by Ethical Committee for Animal Experimentation from the Medical Sector at Université Catholique de Louvain (no. LA1230396).

## Author contributions

Conceptualization: SB and PV. MA-S conducted pilot studies with AdipoRon and initiated this work. Methodology: ND, MS, SB and LN. Formal analysis: ND, MD-LC, MA-S, CS, RV and LN. Investigation: ND, MD-LC and MA-S. Writing original draft: ND, MD-LC, MA-S and SB. Writing review and editing: ND, MD-LC, MA-S, CS, RV, LN, PV and SB. Supervision: SB and PV. Funding acquisition: ND, MA-S, PV and SB. All authors contributed to the article and approved the submitted version.
