# Supplementary material for: Inhibiting the inflammasome with MCC950 counteracts muscle pyroptosis and improves Duchenne muscular dystrophy
Source: Front Immunol. 2022 Dec 7;13:1049076. doi: 10.3389/fimmu.2022.1049076 (PMC9770793; doi:10.3389/fimmu.2022.1049076)
Supplement: Supplementary file 1 [file DataSheet_1.docx]

Supplementary Material

**Table S1. Antibody Information Chart**

| **Antibody** | **Species raised** | **Dilution** | **Incubation time** | **Temp.** | **Product code** | **Source** |
| --- | --- | --- | --- | --- | --- | --- |
| **Immunohistochemistry** | | | | | | |
| CD68 | rabbit polyclonal | 1/500 | 2h30 | RT | ab125212 | Abcam, Cambridge, UK |
| HNE | rabbit polyclonal | 1/200 | Overnight | 4°C | ab46545 | Abcam, Cambridge, UK |
| IL-1β | rabbit polyclonal | 1/200 | 2h30 | RT | ab2105 | Abcam, Cambridge, UK |
| IL-18 | rabbit polyclonal | 1/100 | Overnight | 4°C | ab71495 | Abcam, Cambridge, UK |
| PRDX3 | rabbit polyclonal | 1/1000 | Overnight | 4°C | / | Gift from Bernard Knoops, UCL, Belgiumᵃ |
| TNFα | rabbit polyclonal | 1/100 | 2h30 | RT | ab6671 | Abcam, Cambridge, UK |
| **Immunofluorescence** | | | | | | |
| CD68 | rabbit polyclonal | 1/500 | Overnight | 4°C | ab125212 | Abcam, Cambridge, UK |
| IL-1β | rabbit polyclonal | 1/500 | Overnight | 4°C | ab2105 | Abcam, Cambridge, UK |
| IgG | goat polyclonal | 1/500 | 1h | RT | A-11001 | Invitrogen, Carlsbad, US |
| Laminin-2 (α-2 chain) | rat monoclonal | 1/1000 | Overnight | 4°C | L0663 | Sigma Aldrich, Missouri, USA |
| Myh3 | rabbit polyclonal | 1/200 | Overnight | 4°C | LS-C336252 | LS Bio, Seattle, USA |
| N-GSDMD | rabbit monoclonal | 1/50 | Overnight | 4°C | ab2015203 | Abcam, Cambridge, UK |
| **Western Blotting** | | | | | | |
| Caspase-1 | rabbit monoclonal | 1/5000 | Overnight | 4°C | LS-C138140-100 | Bio-connect, Toronto, Canada |

ᵃLeyens G, Donnay I, Knoops B. Cloning of Bovine Peroxiredoxins-Gene Expression in Bovine Tissues and Amino Acid Sequence Comparison with Rat, Mouse and Primate Peroxiredoxins. *Comp Biochem Physiol B Biochem Mol Biol* (2003) 136(4):943-55. Epub 2003/12/10. doi: 10.1016/s1096-4959(03)00290-2. RT: Room temperature; N-GSDMD: N-Gasdermin D.

**Table S2. Sequences of real-time PCR primers**

| Gene |  |  | Sequence (5’-3’) | Melting temperature |
| --- | --- | --- | --- | --- |
| TGFβ | mouse | Forward | TTGCTTCAGCTCCACAGAGA | 62 |
|  |  | Reverse | TGGTTGTAGAGGGCAAGGAC | 62 |
| IL-18 | human | Forward | TGCAGTCTACACAGCTTCGG | 62 |
|  |  | Reverse | GCAGCCATCTTTATTCCTGCG | 62 |


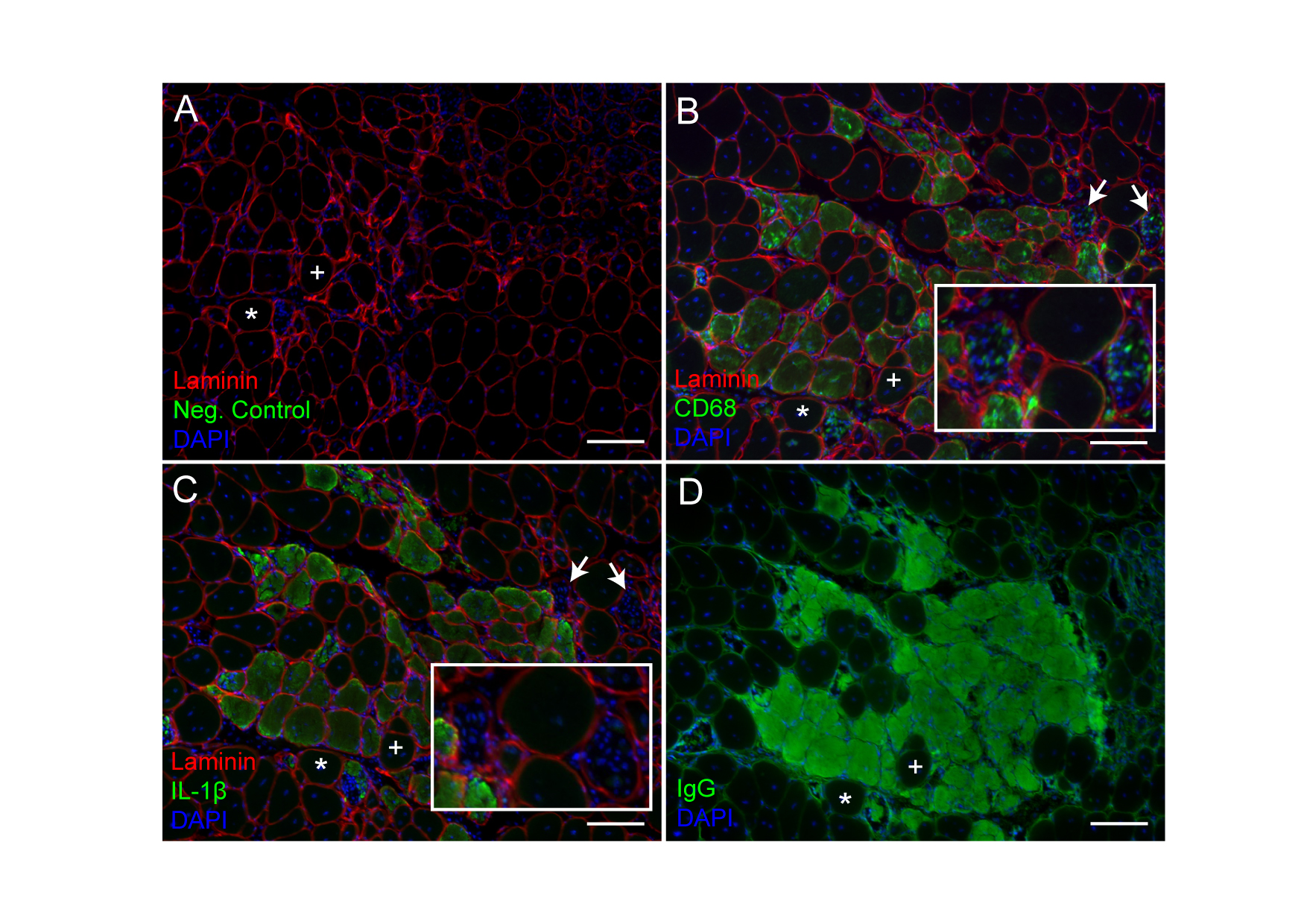


**Fig S1.**  *Negative control of the antibodies to IL-1β and to CD68.* Serial muscle cross sections from mdx mice were incubated overnight without **(A)** or with the rabbit anti-IL-1β **(B)** or rabbit anti-CD68 **(C)** antibody, and then all sections were incubated with the corresponding goat anti-rabbit IgG (H+L)-AF488 secondary antibody. Necrotic fibres were stained with a goat anti-mouse IgG-AF488 (no secondary antibody needed) **(D)**. Applying the same exposure time, no green signal could be distinguished on section **A** incubated only with goat anti-rabbit IgG secondary antibody, showing that this secondary antibody did not yield any unspecific staining in necrotic myofibres. No cross reactivity between the antibodies was observed. The asterisk and plus sign show the same fibres in all the sections. Scale bar = 100 μm.

**
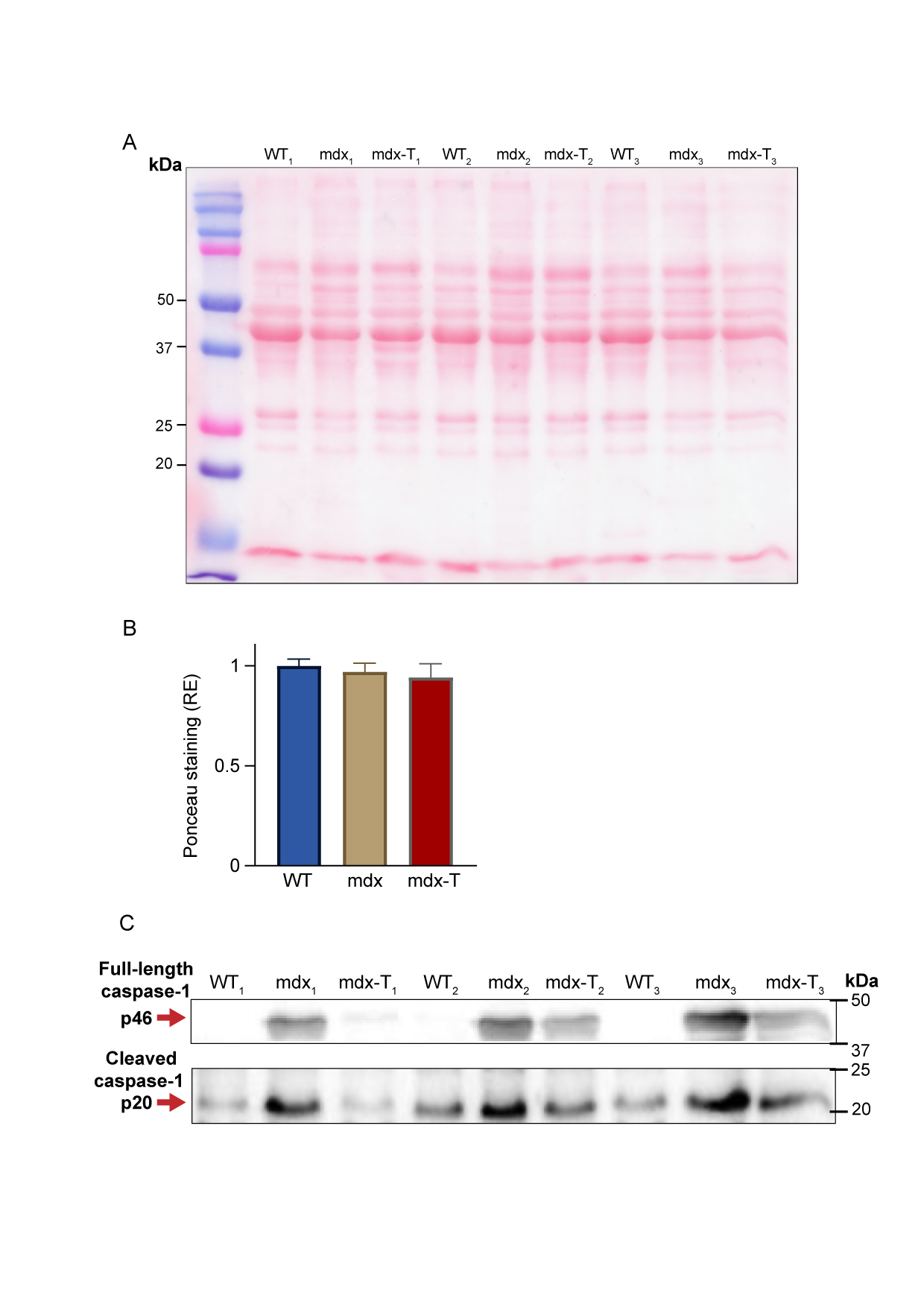
**

**Fig S2.** *Representative Western blotting of full-length caspase-1 (p46) and cleaved caspase-1 (p20).* **(A)** The full ponceau-staining membrane from corresponding Western blotting in WT, mdx and mdx-T mice (three animals for each condition but representative for all the animals studied). **(B)** Quantification of ponceau-staining expressed as relative expression compared to WT. RE: relative expression. **(C)** Western blotting of full-length caspase-1 (p46) and cleaved caspase-1 (p20) after low and high exposure times, respectively.


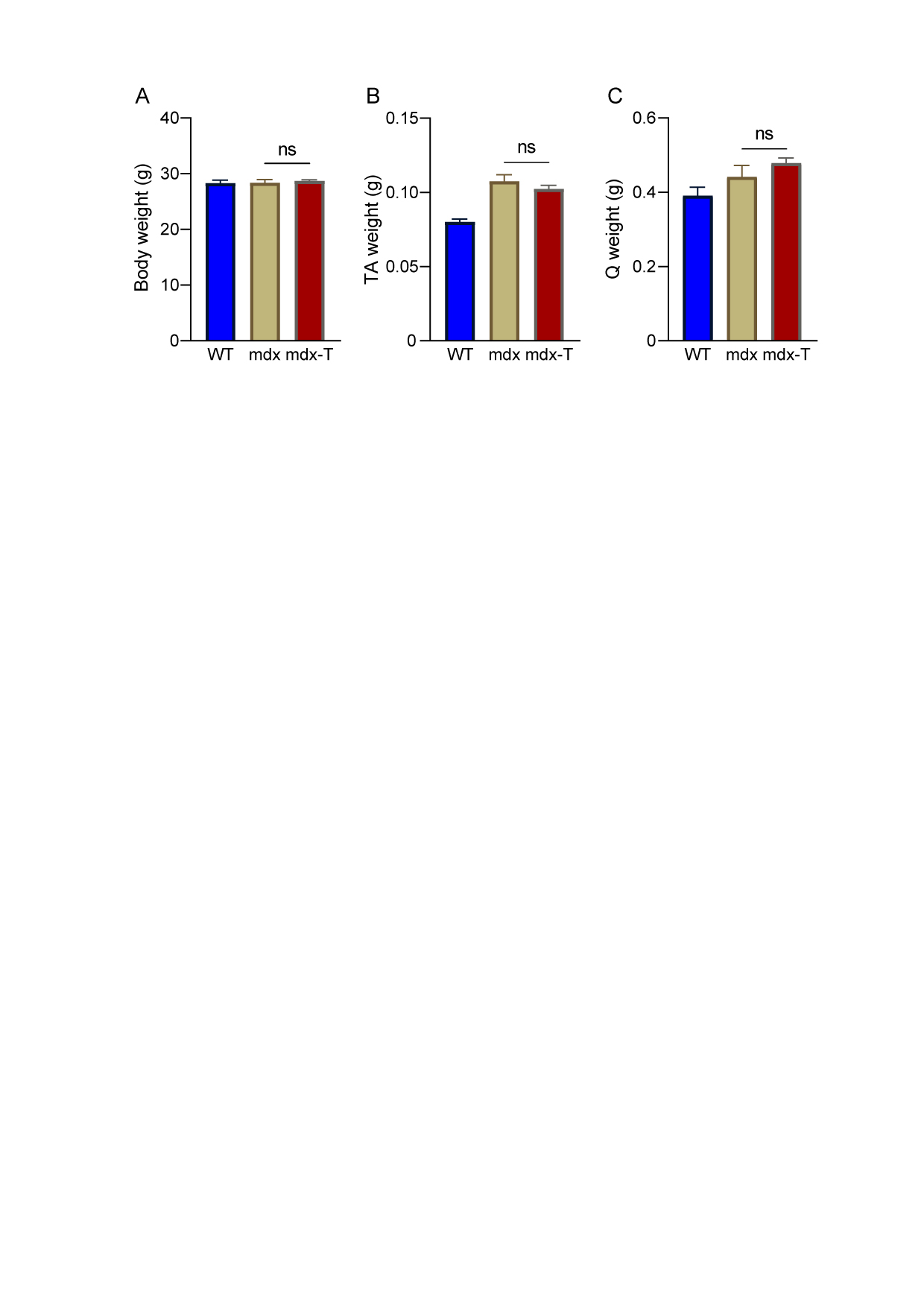


**Fig S3.** *Comparison of body and muscle weights from the three groups of mice.* Three groups of mice were compared at the age of 12 weeks: wild-type (WT) mice, mdx-T mice (mdx mice treated with MCC950), and their mdx littermates (true controls). Mice body weight **(A)** and weights of the tibialis anterior **(B)** and the quadriceps muscle **(C)** were recorded at sacrifice. No difference of body weights among three groups was observed. As previously described, muscle weights from mdx mice were slightly higher than WT (Abou-Samra et al., 2015, ref n°22 in our manuscript) but no difference was observed between mdx and mdx-T animals. Data are means ± SEM; *n* = 6 mice per group for all experiments. Statistical analysis was performed using one-way ANOVA followed by Tukey's test. TA = tibialis anterior, Q = quadriceps, ns = not significant.


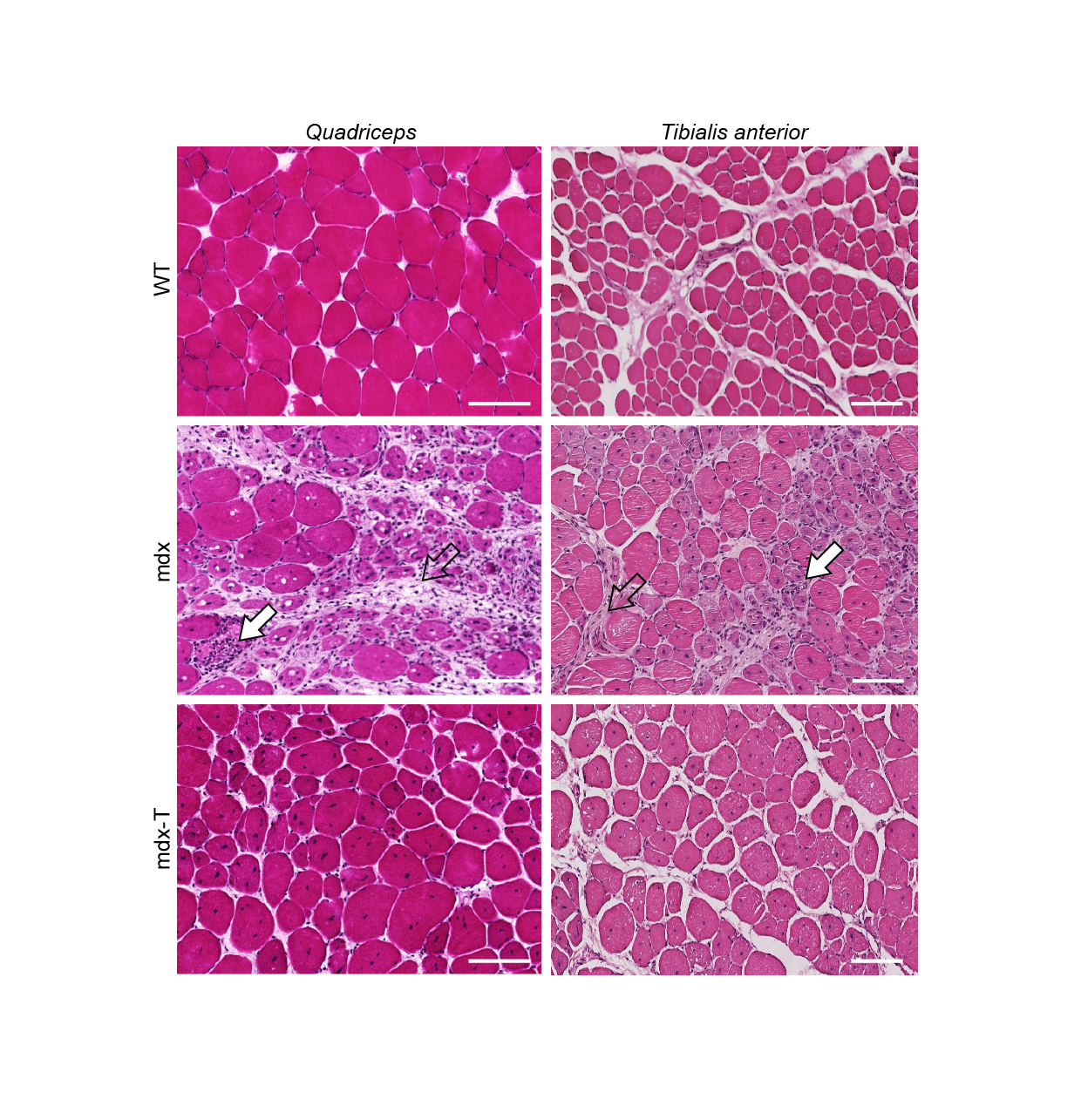


**Fig S4.** *Haematoxylin & Eosin staining of Quadriceps and Tibialis anterior sections from the three groups of mice.* The effects of MCC950 were already observed after H&E staining of TA and Q muscle cross sections. Indeed, while WT muscle cross-section displayed normal morphology, mdx Q and TA showed a dystrophic pattern, witnessed by central nuclei, the presence of fibrotic tissue (empty arrow) and higher degree of inflammatory cell infiltration (white arrow). This phenotype was partially rescued in mdx-T mice. Scale bar = 100 μm.


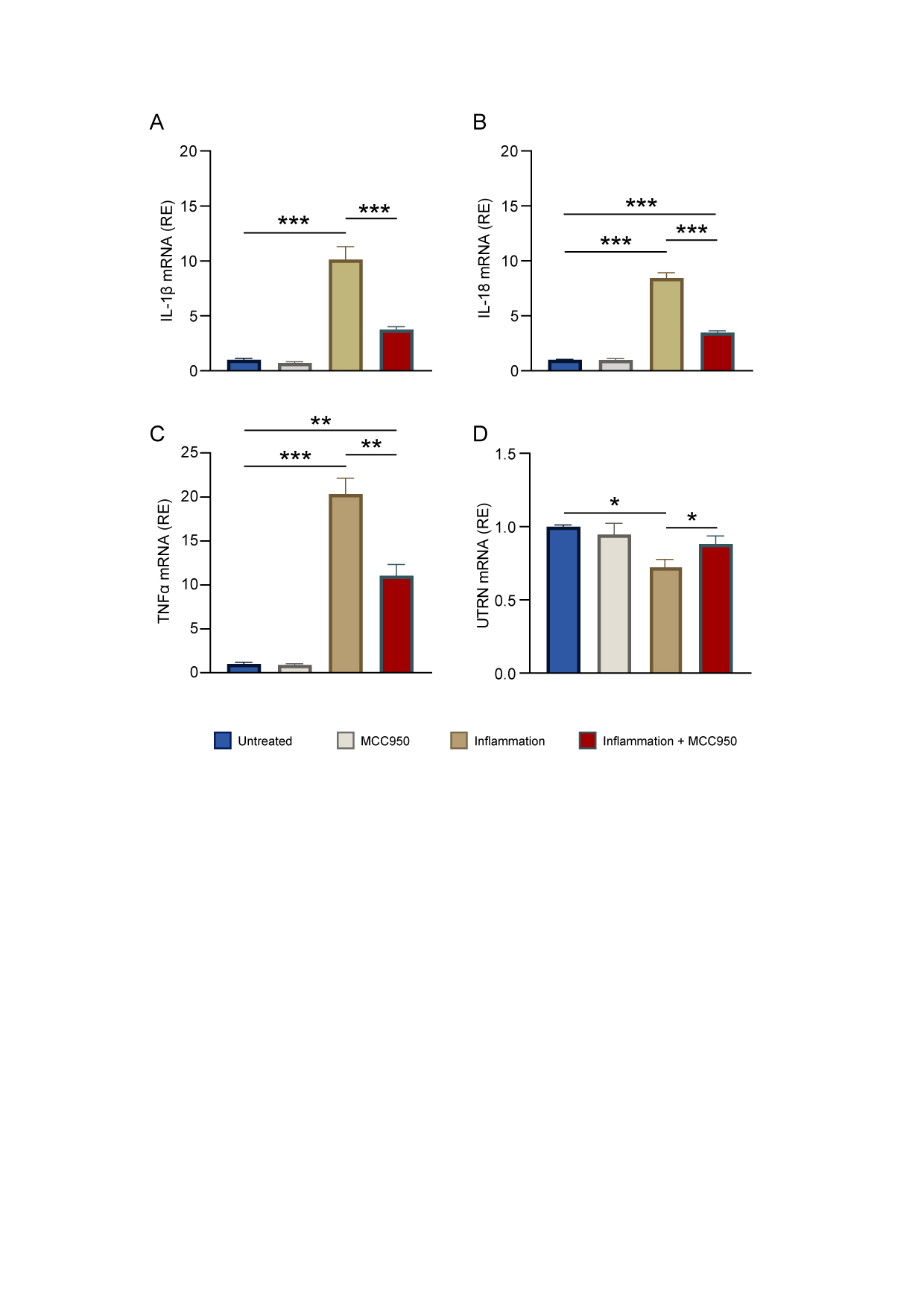


**Fig S5.** *Effects of MCC950 treatment on healthy human myotubes challenged by pro-inflammatory cytokines.* mRNA levels of IL-1β **(A)**, IL-18 **(B)**, TNFα **(C)**, and utrophin A **(D)** in primary culture of human healthy myotubes. Cells were pre-treated or not with MCC950 (10 μM) for 24 h, while being challenged or not with human recombinant TNFα (15 ng/mL) + IFNγ (15 ng/mL) for the last 22 h, and then stimulated with ATP (5mM) for the last 2 h. mRNA levels were normalized to human TATA box-binding protein. The subsequent ratios are presented as relative expression compared with basal conditions (i.e. no inflammation and no MCC950, represented by blue columns). Data are means ± SEM for 3 primary cell cultures, each obtained from a different donor (i.e., 3 healthy subjects). Statistical analysis was performed on paired data using two-way analysis of variance followed by Sidak’s multiple comparison test. ^*^P < 0.05, ^**^P < 0.01, ^***^P < 0.001.
